# Acclimation of *E* *miliania huxleyi* (1516) to nutrient limitation involves precise modification of the proteome to scavenge alternative sources of N and P

**DOI:** 10.1111/1462-2920.12957

**Published:** 2015-08-17

**Authors:** Boyd A. McKew, Gergana Metodieva, Christine A. Raines, Metodi V. Metodiev, Richard J. Geider

**Affiliations:** ^1^ School of Biological SciencesUniversity of EssexWivenhoe Park, WivenhoeColchesterCO4 3SQUK

## Abstract

Limitation of marine primary production by the availability of nitrogen or phosphorus is common. *E* *miliania huxleyi*, a ubiquitous phytoplankter that plays key roles in primary production, calcium carbonate precipitation and production of dimethyl sulfide, often blooms in mid‐latitude at the beginning of summer when inorganic nutrient concentrations are low. To understand physiological mechanisms that allow such blooms, we examined how the proteome of *E* *. huxleyi* (strain 1516) responds to N and P limitation. We observed modest changes in much of the proteome despite large physiological changes (e.g. cellular biomass, C, N and P) associated with nutrient limitation of growth rate. Acclimation to nutrient limitation did however involve significant increases in the abundance of transporters for ammonium and nitrate under N limitation and for phosphate under P limitation. More notable were large increases in proteins involved in the acquisition of organic forms of N and P, including urea and amino acid/polyamine transporters and numerous C‐N hydrolases under N limitation and a large upregulation of alkaline phosphatase under P limitation. This highly targeted reorganization of the proteome towards scavenging organic forms of macronutrients gives unique insight into the molecular mechanisms that underpin how *E* *. huxleyi* has found its niche to bloom in surface waters depleted of inorganic nutrients.

## Introduction

Marine phytoplankton require the essential macronutrients C, N and P for biosynthesis, which on average are required in the molar ratio described by Redfield of C_106_ : N_16_ : P_1_ (Moore *et al*., 2013). Nitrogen is required for the biosynthesis of proteins, nucleic acids and other macromolecules including chlorophyll, as well as low molecular weight compounds such as glycine‐betaine. Phosphorus is required for the synthesis of metabolites, including those involved in energy transfer, phospholipids for membrane synthesis and nucleotides for nucleic acid synthesis.

Living in an environment with an abundance of dissolved inorganic carbon, it is typically the macronutrients N or P that limit or co‐limit growth in regions where resupply by vertical mixing is relatively slow (Moore *et al*., 2008; 2013), whereas the micronutrient Fe is often limiting where macronutrient supplies are high (Moore *et al*., 2006; 2013). Nitrogen availability tends to limit productivity throughout much of the surface low‐ and mid‐latitude ocean at least seasonally (Moore *et al*., 2013), although some regions such as North Atlantic are very close to N and P co‐limitation (Graziano *et al*., 1996; Moore *et al*., 2006), whereas P limitation may be restricted, for example to the eastern Mediterranean Sea (Thingstad *et al*., 2005) or to those organisms that are capable of fixing N_2_ (Moore *et al*., 2013).

In the oceans, dissolved inorganic nitrogen exists in various oxidation states (NH_4_^+^, NO_2_^−^, NO_3_^−^) and is converted and removed by biological activity, while inorganic P exists in one oxidation state (orthophosphate, PO_4_^3−^) that does not undergo biological reduction or oxidation. As well as inorganic nutrients, some phytoplankton utilize dissolved organic N (DON), which consists of compounds such as urea, amino acids, amides, methylamines and purines, and dissolved organic P (DOP), which exists in a many forms of P‐esters or phosphonates. Seasonal differences in nutrient availability occur, with lower concentrations of dissolved inorganic nutrients and higher concentrations of DON and DOP in summer.

One phytoplankter that has evolved to thrive under nutrient‐limited conditions is the ubiquitous coccolithophore *Emiliania huxleyi* found throughout the ocean except the polar regions. *Emiliania huxleyi* is the most abundant coccolithophore (Paasche, 2001), and is important in marine food webs and plays very significant roles in ocean biogeochemistry through its contributions to both primary production via the incorporation of CO_2_ into organic matter and production of CaCO_3_ coccolith plates that surround the cell, which after death and sedimentation contribute to the production of chalk and limestone sediments. The importance of this species led to it being the first haptophyte to have its genome sequenced (Read *et al*., 2013).

*Emiliania huxleyi* can bloom to high concentrations covering vast areas in excess of 200 000 km^2^ (Holligan *et al*., 1993; Sukhanova and Flint, 1998) that are easily detected by satellite remote sensing due to high reflectance of solar radiation by shed coccoliths (Brown and Yoder, 1994). Such blooms are typically found when the mixed surface layers are exposed to both high light and declining macronutrients (Iglesias‐Rodriguez *et al*., 2002). Blooms of *E. huxleyi* often occur after the demise of diatom blooms when NO_3_^−^, PO_4_^3−^ and silicate have been depleted (Holligan *et al*., 1993). It appears that factors that contribute to its success [other than the ability to tolerate high light and a wide range of photon flux densities (PFDs); Suggett *et al*., 2007; McKew *et al*. 2013a] are its ability to thrive on numerous forms of DON (Antia *et al*., 1975; Ietswaart *et al*., 1994; Palenik and Henson, 1997), its very high affinity for inorganic P (Reigman *et al*., 2000) and its ability to utilize DOP (Dyhrman and Palenik, 2003).

The ability of *E. huxleyi* to thrive in high light experienced during blooms involves large changes in the components of the proteome that allowed cells to alter their capacities to absorb light and resist photooxidative stress, but relatively small changes in the remainder of the proteome (McKew *et al*., 2013a, 2013b). In this present study, we used liquid chromatography‐tandem mass spectrometry (LC‐MS/MS) shotgun proteomics to investigate readjustment of the proteome in response to both N and P limitation. The most notable responses were in the abundance of proteins associated with nutrient assimilation, giving a unique, mechanistic insight into how *E. huxleyi* acclimates to low‐nutrient environments.

## Results and discussion

**Figure 1:**
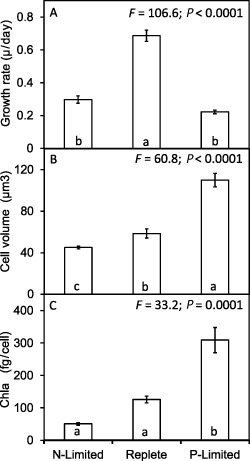
Growth rates of *E* *miliania huxleyi* 1516 and changes in cellular biovolume and Chl *a* content in response to N and P limitation. Significant differences are shown by analysis of variance and Tukey honestly significant difference tests (different letters a, b, c indicate significant differences at 0.05 confidence level).

**Figure 2:**
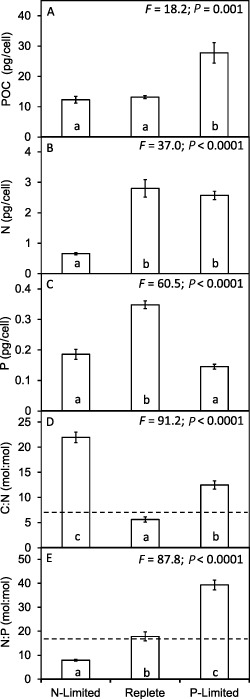
Changes in cellular POC, N and P content of *E* *miliania huxleyi* 1516 and shifts in molar stoichiometry of POC : N : P in response to N and P limitation. Significant differences are shown by analysis of variance and Tukey honestly significant difference tests (different letters a, b, c indicate significant differences at 0.05 confidence level). Dotted lines on D and E represent Redfield ratio stoichiometry.

Differences in the steady‐state growth rates between nutrient‐replete, P‐limited and N‐limited conditions were accompanied by differences in cell size and elemental composition (Figs 1 and 2). Relative to nutrient‐replete cells, there was a significant decrease in cellular biovolume of N‐limited cells, and in contrast a significant increase in the biovolume of the P‐limited cells (Fig. 1B). Significant differences in cellular Chl *a* content were observed between the three treatments, being lowest in N‐limited and highest in P‐limited cells (Fig. 1C). Both N‐limited and nutrient‐replete cells contained about 50% less particulate organic carbon (POC) than the larger P‐limited cells (Fig. 2A), consistent with previous reports (Reigman *et al*., 2000). Cellular biovolume was highly correlated with cellular POC content (r = 0.92; *P* < 0.0001) and particulate inorganic carbon (PIC) content (r = 0.89; *P* < 0.0001), less so with cellular N content (r = 0. 61; *P* = 0.045), but not with cellular phosphorus (r = −0.36; *P* = *ns*). Particulate inorganic carbon followed the same pattern as POC, and consequently the PIC : POC ratios did not vary significantly among the three conditions (PIC : POC nutrient‐replete 0.56 ± 0.10; N‐limited 0.70 ± 0.02; P‐limited 0.73 ± 0.10).

While the P‐limited cells contained high levels of POC, they contained less than half the particulate P content of the replete cells (Fig. 2C), and while the N‐limited cells contained similar levels of POC to the replete cells the particulate N was some fourfold lower (Fig. 2B). The resulting molar ratios of cellular POC : N (Fig. 2D) and N : P (Fig. 2E) were also significantly different. The POC : N : P observed in the nutrient‐replete cells (C_98_ : N_18_ : P_1_) was similar to Redfield stoichiometry (C_106_ : N_16_ : P_1_). Significantly higher POC : N ratios were observed in the P‐limited (POC : N = 12.5 mole/mole) and N‐limited cultures (POC : N = 22) than in nutrient‐replete (POC : N = 5.6). The increase of POC : N in response to P limitation was primarily due to increase in cell size and POC, whereas the increase in response to N limitation was brought about by lower N. Relative to the nutrient‐replete cells, which had an N : P ratio of 17.8, the N‐limited cells had a significantly lower N : P ratio of 7.9, whereas P‐limited cells had a significantly higher ratio of 39. These deviations from Redfield C : N : P stoichiometry that occurred in response to nutrient limitation are consistent with previous observations for *E. huxleyi* (Leonardos and Geider, 2005; Borchard *et al*., 2011), and are consistent with N limitation placing a constraint on protein synthesis, while P limitation constrains RNA production (Loladze and Elser, 2011).

NH_4_^+^ was the preferred nitrogen source (confirmed by daily measurements of residual NO_3_^−^ and NH_4_^+^) and NO_3_^−^ was consumed only after NH_4_^+^ had been depleted. This was also confirmed by nutrient uptake assays that involved incubating N‐limited cells in NO_3_^−^‐replete, NH_4_^+^‐replete and NH_4_^+^NO_3_^−^‐replete media and measuring N uptake at hourly intervals. V_max_ was 2.8‐fold greater when supplied NH_4_^+^ (6.86 ± 0.18 fmol N cell^–1^ hr^–1^) compared with NO_3_^−^ (2.43 ± 0.18 fmol N cell^–1^ hr^–1^), and when supplied NH_4_^+^NO_3_^−^ only NH_4_^+^ uptake was observed.

**Figure 3:**
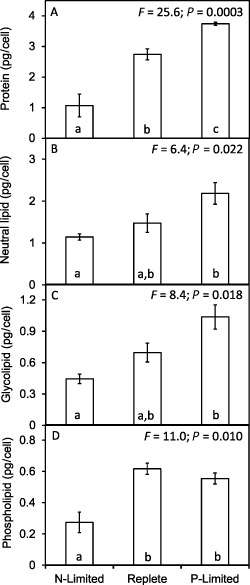
Changes in cellular protein and lipid contents of *E* *miliania huxleyi* 1516 in response to N and P limitation. Significant differences are shown by analysis of variance and Tukey honestly significant difference tests (different letters a, b, c indicate significant differences at 0.05 confidence level).

Cellular protein followed the same pattern as, and correlated with, cellular biovolume (r = 0.84; *P* = 0.001) and cellular N (r = 0.78; *P* = 0.005), where relative to the nutrient‐replete cells protein content per cell was significantly lower in the smaller N‐limited cells and significantly higher in the larger P‐limited cells (Fig. 3A). While the relative differences in protein content per cell between the treatments are realistic, the absolute cellular protein contents (e.g. approximately 3 pg cell^–1^ under nutrient‐replete conditions) appear to be low given the observed cellular N contents. We may have underestimated cellular protein either due to incomplete protein extraction and/or differences in sensitivity of the protein assay between *E. huxleyi* proteins and the BSA standards. However, we also note that the reported values are in line with other studies (e.g. Strom *et al*., 2003; Kaffes *et al*., 2010), suggesting underestimation of protein content of this organism may be common. RNA content per cell was two to three times greater in the nutrient‐replete cells than the P‐limited and N‐limited cells (data not shown), giving similar protein : RNA ratios under replete and N‐limited conditions but a significantly higher ratio under P limitation.

The lowest concentrations of neutral lipid (Fig. 3B), glycolipid (Fig. 3C) and phospholipid (Fig. 3D) were observed in the N‐limited cells, with significantly higher levels present in the P‐limited cells (approximately twofold higher in all cases). Intermediate concentrations of neutral lipids and glycolipids were present in the nutrient‐replete cells. Phospholipids did not follow this pattern as slightly higher levels were measured in the nutrient‐replete than in P‐limited cells, although the difference was not significant. Neutral lipids represented 53% of total lipid in the nutrient‐replete cells, and 61% and 58% of total lipid in the N‐limited and P‐limited cells respectively. Glycolipid as a percentage of total lipid was similar in all treatments (24% N‐limited, 25% nutrient‐replete and 27% in P‐limited cells). Phospholipid represented 22% of total lipid in the nutrient‐replete cells and 15% under both nutrient‐limited conditions. While the abundance of the lipid fractions differed between treatments, the ratios of the constituent fatty acids were highly similar. For example, docosahexaenoic acid (DHA C22:6n3) was the most abundant lipid, accounting for 34–35% of total lipid in all three treatments. Other abundant lipids were myristic acid (C14:00), palmitic acid (C16:00), oleic acid (C18:1n9c), linolenic acid (C18:3n3), docosatetraenoic acid (C22:4n6) and eicosapentaenoic acid (C20:5n3), indicating that while cellular contents of lipids changed significantly the fatty acid composition of the lipids remained highly similar. The observed lipid composition was similar to previously reported (Pond and Harris, 1996; Sayanova *et al*., 2011).

Neutral lipids are present in all growth phases of *E*. *huxleyi*, but these energy storage lipids have been shown to increase particularly during N‐starved stationary phase (Pond and Harris, 1996) as carbon incorporation into lipids is 40–60%, whereas carbon incorporation into protein is lower at 20% due to the high N demand (Fernández *et al*., 1994). It has been shown that increases in neutral lipid are observed 4–10 days after nutrient stress under both N and P limitation (e.g. Eltgroth *et al*., 2005). We also observe this response in N‐limited cultures in the early stage of N limitation (data not shown), but here we show that during steady‐state growth (cultures were in balanced growth and had been under nutrient limitation for 1 month before sampling) the levels of cellular lipid return to lower levels are in fact the lowest in the smaller N‐limited cells. However, the fact that protein was also much lower under N limitation is evident in the lipid : protein ratio (1 under replete and P‐limited conditions increasing to 1.8 under N limitation) as lipid represents a non‐nitrogenous pool. Lipid responses are also seen under P limitation, as many phytoplankton, including *E. huxleyi*, can reduce their cellular phosphorus requirements by substituting non‐phosphorus membrane sulfolipids for phospholipids (Van Mooy *et al*., 2009), and we observed that the phospholipid pool did not follow the same pattern as glyco‐ and neutral lipids of being higher in the larger P‐limited cells. Although lipid increased under P limitation, lipid represented 20% of cell C under replete, decreasing to 15% and 14% under N and P limitation respectively.

### Proteomic analysis

Three technical replicates were run for each of the four biological replicates of each of the N‐limited, P‐limited and nutrient‐replete conditions, resulting in 613 505 total scans, of which 265 368 spectra were confidently assigned to 3012 different proteins. Spectral counts ranged from 1 to 6418 per protein, with over 500 proteins having 100 or more spectral counts assigned. Of the 500 proteins with the highest number of spectral counts, 36 were at least twofold higher under N limitation (relative to replete) and 53 were at least twofold higher under P limitation. Similar numbers of proteins were also twofold less abundant (52 under N limitation and 61 under P limitation, both relative to nutrient‐replete). Many proteins were detected with too few spectral counts to allow comparison among treatments. Approximately 2000 of the detected proteins had very low spectral counts, averaging 1 or less per run. Thus, it was necessary to sum the spectral counts for related proteins at the level of macromolecular complexes (e.g. chloroplast ATP synthase) or metabolic pathways (e.g. glycolysis) or by function (e.g. amino acid metabolism) to obtain meaningful information on changes of abundance among treatments (Table 1; for details of all individual proteins assigned to particular functions or pathways, see Table S1). Between 13% and 15% of the spectral counts were assigned to proteins with unknown functions.

**Table 1:** Differences in abundance of normalized spectral counts assigned to proteins associated with specific functions or pathways

	Spectral counts					Tukey HSD		
	N‐Limited	Replete	P‐Limited	*F* (ANOVA)	*P* (ANOVA)	N‐Limited	Replete	P‐Limited
Light harvesting	329	338	455	28	< 0.001	b	b	a
Photosynthetic electron transfer chain	152	158	156	0.3	0.782	a	a	a
Chloroplast ATP synthase	321	343	330	2.7	0.131	a	a	a
Calvin cycle	338	392	375	4.4	0.052	b	a	ab
Other photosynthesis	26	52	31	42	< 0.0001	b	a	b
Carotenoids/porphyrin/pigment synthesis	39	79	54	36	0.0001	c	a	b
Thioredoxin	2.6	1.4	3.5	32	< 0.001	b	c	a
FtSH	8.8	14	9.0	27	< 0.001	b	a	b
Bicarbonate transport	2.7	3.1	0.8	9.7	0.007	a	a	b
Carbonic anhydrase	3.9	6.2	5.0	1.9	0.213	a	a	a
Glycolysis	173	148	208	6.5	0.021	ab	b	a
Krebs cycle	127	101	129	7.2	0.016	a	b	a
Other energy production and conversion	123	97	113	6.9	0.018	a	b	ab
Amino acid metabolism	137	174	146	28	< 0.001	b	a	b
Nucleotide metabolism	59	63	56	1.9	0.210	a	a	a
Fatty acid synthesis	119	130	123	4.5	0.049	b	a	ab
Fatty acid degradation beta oxidation	29	32	30	0.9	0.453	a	a	a
Carbohydrate metabolism	81	71	62	7.9	0.013	a	ab	b
Isoprenoid biosynthesis	7.2	14	13	20	0.001	b	a	a
Other secondary metabolism	110	122	94	11	0.005	a	a	a
Heat shock proteins HSP90	46	63	51	13	0.003	b	a	b
Heat shock proteins HSP70	100	108	100	1.4	0.298	a	a	a
Heat shock proteins HSP60	22	36	33	20	0.001	b	a	a
Heat shock proteins HSP40 (DnaJ)	11	13	8	8.6	0.010	ab	a	b
Heat shock proteins HSP TCP‐1	3.6	5.3	3.3	5.0	0.040	ab	a	b
Clp protease	22	27	19	8.2	0.012	ab	a	b
Peroxidase	14	7.1	9.8	9.6	0.007	a	b	ab
Vitamin B6 synthesis	0.7	2.5	2.0	5.2	0.036	b	a	ab
Superoxide dismutase	3.4	2.6	3.2	0.6	0.561	a	a	a
Myosin and actin	34	17	29	13	0.003	a	b	a
Microtubules	10	16	7.1	17	0.001	b	a	b
Cell cycle	44	43	36	8.1	0.012	a	a	b
Ribosomal proteins	204	241	144	19	0.001	a	a	b
Chloroplast ribosomal proteins	35	60	15	41	< 0.0001	b	a	c
Histones	164	108	60	97	< 0.0001	a	b	c
Translation/transcription	309	356	260	17	0.001	ab	a	b
Protein modification and transport	350	302	315	3.5	0.083	a	a	a
Protein degradation (ubiquitin and proteasome)	127	94	99	16	0.001	a	b	b
Signal transduction	31	17	21	12	0.004	a	b	b
Urea cycle	12	30	16	26	< 0.001	b	a	b
Nitrogen assimilation/metabolism	100	41	40	335	< 0.0001	a	b	b
Phosphorous assimilation/metabolism	25	6	267	37	< 0.0001	b	b	a
Sulfate assimilation/metabolism	47	57	59	11	0.006	b	a	a
Unknown/unassigned function	690	602	603	4.9	0.040	a	a	a

Significant differences are shown by analysis of variance and Tukey honestly significant difference tests (different letters a, b, c indicate significant differences at 0.05 confidence level).

### Nitrogen acquisition

**Figure 4:**
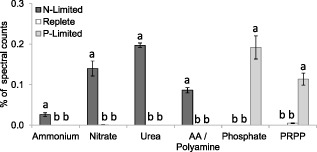
Changes in relative abundance of nutrient transporter proteins (for nutrients as labelled: AA, amino acid; PRPP, phosphate repressible phosphate permease) in *E* *miliania huxleyi* 1516 in response to N and P limitation. Significant differences are shown by analysis of variance (*P* < 0.0001 in all cases) and Tukey honestly significant difference tests (different letters a, b, c indicate significant differences at 0.05 confidence level).

**Figure 5:**
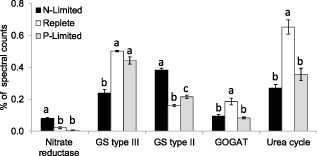
Changes in relative abundance of nitrate reductase, the GS/GOGAT (glutamine synthetase‐glutamate synthase) and urea cycle pathway proteins in *E* *miliania huxleyi* 1516 in response to N and P limitation. Significant differences are shown by analysis of variance (*P* < 0.002 in all cases) and Tukey honestly significant difference tests (different letters a, b, c indicate significant differences at 0.05 confidence level).

Some of the most significantly upregulated proteins in response to nutrient limitation were those involved in nitrogen and phosphorus transport (Fig. 4). Numerous nitrogen transporters were detected in the N‐limited cells, but not in the replete and P‐limited conditions. Despite the fact that NH_4_^+^ was the preferred N source, and that NO_3_^−^ was not drawn down to any appreciable extent in nutrient‐replete or P‐limited treatments, NH_4_^+^ transporters were only detected under N limitation. This suggests that the abundance of NH_4_^+^ transporters under N‐replete conditions remains below the limit of detection of the shotgun proteomic approach, but that significant upregulation during N limitation allowed these transporters to be detected. The abundance of NO_3_^−^ transporters (Fig. 4) and nitrate reductase (Fig. 5) was significantly greater under N limitation, consistent with the preference of *E. huxleyi* for NH_4_^+^ relative to NO_3_^−^. This suggests nitrate reductase was induced in the N‐limited cells as all available NH_4_^+^ was continuously assimilated, and thus 50% of their N uptake was from NO_3_‐, but under both P‐limited and nutrient‐replete conditions nitrate reductase was repressed due to abundant available NH_4_^+^. Reduced expression of nitrate reductase when *E. huxleyi* was grown on NH_4_^+^ has been reported previously (Bruhn *et al*., 2010), while in diatoms both NO_3_^−^ reductase transcription (Song and Ward, 2004) and enzyme activity (Lomas and Gilbert, 1999) have been shown to be induced by NO_3_^−^ and inhibited by NH_4_^+^.

**Figure 6:**
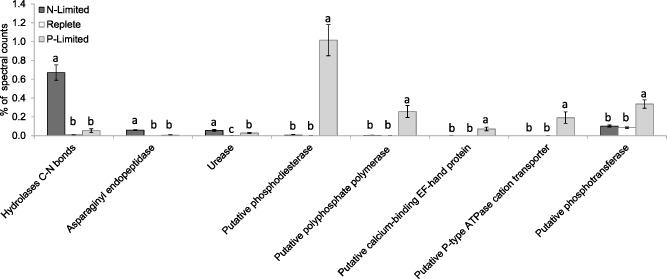
Changes in relative abundance of other proteins associated with organic N and P turnover/assimilation in *E* *miliania huxleyi* 1516 in response to N and P limitation. Significant differences are shown by analysis of variance (*P* < 0.01 in all cases) and Tukey honestly significant difference tests (different letters a, b, c indicate significant differences at 0.05 confidence level).

As well as the observed upregulation of inorganic nitrogen transporters under N limitation, the proteome was further modified for the uptake of DON. Urea and amino acid/polyamine transporters were observed in N‐limited cells, but were undetected in nutrient‐replete and P‐limited cells (Fig. 4). Urease, the enzyme that catalyses the hydrolysis of urea into carbon dioxide and NH_4_^+^, was upregulated alongside the urea transporters (Fig. 6). The upregulation of all these inorganic and organic N transporters strongly suggests a strategy for scavenging all types of N sources from the surrounding environment, which is induced by N limitation. Numerous C‐N hydrolases were upregulated to an even greater extent (Fig. 6); the most abundantly detected of these was AMI1 formamidase (hydrolyses formamide into formate and NH_4_^+^). This was despite the fact that the only form of N provided in our experiments was NH_4_NO_3_, indicating that expression was induced by N limitation not by the presence of urea or formamide. While it is possible that further regulation of expression of key genes may be brought about by the presence of organic compounds such as urea or formamide, it has previously been shown that large fold changes in formamidase expression are approximately equal when *E. huxleyi* is growing on formamide or under N limitation, when compared with growth under NO_3_‐replete conditions (Bruhn *et al*., 2010). Cytosolic hydrolases such as formamidase are upregulated when growing on amides transported from the environment, but a number of different hydrolases were upregulated, the exact functions of which are unknown, so it is possible that some may also play a role in internal remobilization of N from amino acids, proteins or other N containing molecules to provide glutamine synthetase (GS) with NH_4_^+^ under N limitation. Although internal remobilization may contribute to changes in cell composition when growth is unbalanced, such as what occurs when cells enter stationary phase in nutrient‐starved cultures, our cultures were in balanced growth, for which we do not expect internal remobilization to be significant. This is because internal remobilization would require that the molecule of interest be synthesized and then subsequently degraded without making a contribution to net growth; as such, it would represent a metabolic cost without an obvious benefit.

Our observations are consistent with previous reports that *E. huxleyi* can thrive on various forms of DON, including urea, purines, and amides such as acetamide and formamide (Antia *et al*., 1975; Ietswaart *et al*., 1994; Palenik and Henson, 1997; Bruhn *et al*., 2010). Formamidase activity has been shown to increase up to 31‐fold during N limitation, but formamidase expression is reduced when growing on NO_3_^−^ (Bruhn *et al*., 2010). While some other phytoplankton can also utilize DON (Wawrik *et al*., 2009), some organic sources of N such as formamide have been shown to be a poor N source for numerous phytoplankton, with many species unable to grow on it at all (Palenik and Henson, 1997). In contrast, for *E. huxleyi*, formamide was in fact a significantly better N source than NO_3_^−^. Asparaginyl endopeptidase was also significantly more abundant under N limitation (Fig. 6) and may play a role in proteolysis and mobilization of N within proteins, as can occur in vacuolar bulk protein degradation in plants (Chen *et al*., 2004).

### Phosphorus acquisition

**Figure 7:**
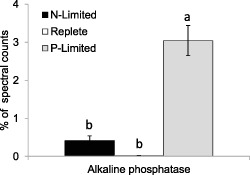
Large change in relative abundance of alkaline phosphatase enzymes (for dephosphorylation of organic P) in *E* *miliania huxleyi* 1516 in response to N and P limitation. Significant differences are shown by analysis of variance (*P* < 0.0001) and Tukey honestly significant difference tests (different letters a and b indicate significant differences at 0.05 confidence level).

Proteome modification during P limitation was even more pronounced than that observed in response to N limitation. Inorganic PO_4_^3−^ transporters were only detected during P limitation (Fig. 4) as were numerous other proteins with putative roles in P assimilation (Fig. 6). Proteins that were significantly higher under P limitation also included a phosphate repressible phosphate permease (Fig. 4), and putative phosphodiesterase, polyphosphate polymerase, EF‐hand protein, P‐type ATPase cation transporter, and phosphotransferase proteins (Fig. 6). Two hypothetical proteins (4739624 and 463656) detected under P limitation and absent under N limitation and nutrient‐replete conditions may also have a role in phosphorus metabolism. The most striking change in response to P limitation was a large increase in the abundance of alkaline phosphatase (AP), which in terms of spectral counts was the most abundantly detected protein under P limitation (Fig. 7). Alkaline phosphatase cleaves 5′ phosphate groups from DNA and RNA, nucleotides and proteins by hydrolysis of phosphate‐ester bonds. Thus, as in *Aureococcus anophagefferens* (Wurch *et al*., 2011) and *Thalassiosira pseudonana* (Dyhrman *et al*., 2012), acclimation to P limitation in *E. huxleyi* resulted in the upregulation of the ability to obtain inorganic P by cleaving phosphate from dissolved organic matter and enhancing transport of inorganic P into the cell by increasing the abundance of PO_4_^3−^ transporters.

Alkaline phosphatases are cell surface proteins in P‐limited *E. huxleyi* for scavenging external sources of organic P from the environment (Dyhrman and Palenik, 2003; Landry *et al*., 2006). Alkaline phosphatase is expressed by many phytoplankton in response to P limitation (Kuenzler and Perras, 1965; Sakshaug *et al*., 1984; Dyhrman and Palenik, 1997; Dyhrman and Palenik, 2003; Wurch *et al*., 2011), and AP activity has been suggested as an index of P limitation (Beardall *et al*, 2001). However, Kuenzler and Perras (1965) reported *E. huxleyi* had the highest AP production rates of 10 different algae, and Reigman and colleagues (2000) reported *E. huxleyi*'s affinity for P to be the highest recorded for a phytoplankton species and can dominate in competition experiments with low P (Reigman *et al*., 1992). Diatoms in contrast may be poor competitors in low P environments (Egge, 1998). This evidence all suggests how blooms of *E. huxleyi* can occur after demise of diatom blooms when inorganic N and P are depleted, and particularly why it is hypothesized that P limitation is a key fact that allows *E. huxleyi* to outcompete other phytoplankton and bloom to large cell densities (Lessard *et al*., 2005). The AP activity in *E*. *huxleyi* has been shown to be induced approximately 6 days after P limitation, and its activity is rapidly lost when PO_4_^3−^ is added to P‐limited cultures (Dyhrman and Palenik, 2003). Expression of the gene ehap1 has been shown to increase in *E. huxleyi* over 1000‐fold within 24 h after cells were transferred from P‐replete to P‐deplete medium with its expression being regulated by extracellular and intracellular P concentrations (Xu *et al*., 2006).

The thickness of the diffusion boundary layer decreases as cells become smaller, meaning there is less likelihood of limitation of nutrient uptake by molecular diffusion. Thus, the decrease of cell size in *E. huxleyi* in response to N limitation could be interpreted as an acclimation that increases the potential for nutrient uptake. In fact, the reduction of transport limitation with decreasing cell size is commonly invoked as one of the factors that favours small cell size in the oligotrophic ocean. The increase of cell size in response to P limitation in *E. huxleyi* is inconsistent with this tendency and requires another explanation. Cell surface biotinylation experiments have shown that APs are dominant cell surface proteins in P‐limited *E. huxleyi* 1516 (Landry *et al*., 2006). The very large increase in abundance of the EHAP1 protein (433041) as well as another AP (414308), which together accounted for 3% of the spectral counts in P‐limited *E. huxleyi*, will require a large area of plasma membrane for attachment and may explain the increase in cell surface area relative to cell P content under P limitation. The larger P‐limited cells had about 40% more surface area than nutrient‐replete cells, which would be able to accommodate the additional cell surface AP. Some of the differences in cell sizes under N or P limitation could also possibly be attributed to the different forms of nutrient limitation affecting the progression of cells though the cell cycle. For example, if cells are arresting during the G1 phase (before much of the cells growth has occurred) under N limitation, but during the G2 + M phase under P limitation, differences in the average cells sizes of the populations would be seen. In both the diatom *Thalassiosira weissflogii* and the coccolithophorid *Hymenomonas carterae*, N‐starved cells arrested in the early part of the cell cycle at the G1 phase (Vaulot *et al*., 1987). The length of both G1 and G2 phases decrease with growth rate increase in the diatom *Thalassiosira pseudonana* (Claquin *et al*., 2002) under both N and P limitation, but with a lower percentage of cells in the S phase (when DNA is replicated) and a larger percentage of cells in the G2 + M phase under P limitation. In contrast, P starvation of *Prochlorococcus* spp. has been shown to induce accumulation of cells in the S phase as well as in the G2 phase in (Parpais *et al*., 1996).

### N assimilation and amino acid synthesis

Most of the proteins involved in nitrogen assimilation and amino acid synthesis were less abundant in the slower growing nutrient‐limited cells (Fig. 5 and Table 1). Glutamate synthase (GOGAT) and the urea cycle enzymes (carbamoyl‐phosphate synthase (CPS), argininosuccinate synthase and argininosuccinate lyase) were approximately twofold less abundant in the nutrient‐limited conditions (Fig. 5). Glutamate synthase is required for the assimilation of NH_4_^+^ into amino acids, whereas the urea cycle enzyme CPS plays a key role in the synthesis of amido‐N from NH_4_^+^ (Allen *et al*., 2011). The urea cycle also plays a role in the recycling of NH_4_^+^ generated by protein turnover (Armbrust *et al*., 2004). The cycle appears to be essential for diatom growth, with knockdown of CPS found to impair the response of nitrogen‐limited diatoms to nitrogen addition (Allen *et al*., 2011); our data suggest that it also plays a role in the coccolithophore *E. huxleyi*. In contrast, GS, also required for NH_4_^+^ assimilation into amino acids via the GS‐GOGAT pathway, showed a more complicated pattern of response. Significantly, the decrease was evident under both N limitation and P limitation, suggesting that the reduced demand for N due to the lower rates of biosynthesis allowed reallocation of N to other proteins. In addition, under N limitation, the larger type III GS (436026; 72 kDa), which requires considerably more N to synthesize, was significantly less abundant (approximately twofold), whereas smaller type II GS (437187, 470023, 69253, 124007; 14–47 kDa) were upregulated and were approximately twofold more abundant (Fig. 5), although the sum of GS type II and GS type III remained a constant proportion of the spectral counts. Upregulation of the type II GS and nitrate reductase in the N‐limited cultures (where 50% of N uptake was from NO_3_^−^) is consistent with the observation in diatoms of the role of II GS in assimilating NH_4_^+^ derived from NO_3_^−^ reduction (Takabayashi *et al*., 2005).

### Glycolysis and Krebs cycle

The sum of spectral counts assigned to proteins involved in glycolysis were 17% greater under N limitation and 40% more abundant under P limitation. Proteins involved in the Krebs cycle were also some 50% greater in both P‐limited and N‐limited cells than in nutrient‐replete cells. This pattern is consistent with the high ratio of respiration to photosynthesis that is typically observed in nutrient‐limited cells. Isocitrate dehydrogenase was the exception, which was most abundant in nutrient‐replete cells, although the difference between P‐limited and nutrient‐replete conditions was not significant. The higher rate of amino acid synthesis required to support higher growth rates in nutrient‐replete conditions may account for this pattern. Isocitrate dehydrogenase catalyses the synthesis of 2‐oxoglutarate, which is one of the substrates used in amino acid synthesis via the GS‐GOGAT pathway. Also, among the mitochondrial proteins that were twofold less abundant under nutrient limitation was pyruvate carboxylase (B9X0T8), which catalyses the carboxylation of pyruvate to form oxaloacetate (OAA), and thus plays a role in the anapleurotic assimilation of bicarbonate, leading to synthesis of aspartate, other amino acids and pyrimidines.

### Protein synthesis

Relative to nutrient‐replete cells, the sum of proteins involved in transcription and translation was approximately 13% lower under N limitation and 25% lower under P limitation (Table 1). Approximately 300 proteins were assigned to this group, although for most we detected too few spectral counts to determine any differences at the level of individual proteins.

Large differences were evident in abundance of ribosomal proteins, which were 20% less abundant under N limitation and almost 50% lower under P limitation than in nutrient‐replete cultures (Table 1), with greater reductions in the abundance of chloroplastic ribosomal proteins than of cytoplasmic ribosomal proteins. The large reduction under P limitation is consistent with the growth rate hypothesis, which links synthesis of RNA to P assimilation (Sterner and Elser, 2002). Assuming limited variability in the ribosomal efficiency, the growth rate hypothesis predicts proportionality between specific growth rate and rRNA : protein. This hypothesis has also been invoked to explain differences in organismal C : N : P ratios, which are assumed to be caused by differences in the amounts of RNA needed to meet the protein synthesis demands of rapid rates of biomass growth and development (Sterner and Elser, 2002).

### Proteolysis

Total counts for proteins involved in protein degradation (the sum of counts of over 130 individual proteasome and ubiquitin proteins) were higher under N limitation (Table 1). The eukaryotic ubiquitin‐proteasome system plays an essential role in adaptation to N limitation (Sato *et al*., 2011), and the observed upregulation under N limitation suggests an increase in proteins being ubiquitylated for targeted degradation by the proteasome to remobilize nitrogen.

### Histones

Histones are small, basic proteins that interact with the DNA double helix to form nucleosomes (Mcghee and Felsenfeld, 1980). Histones 2A and 2B differed significantly among treatments (Table 1). Relative to the nutrient‐replete cells, these proteins were 50% more abundant under N limitation and 44% less abundant under P limitation. The proportion of the proteome assigned to histones followed a similar pattern to DNA‐to‐protein ratio (not shown), being highest in N‐limited cells, intermediate in nutrient‐replete cells and lowest in P‐limited cells.

## Conclusions

In this study we observed modest changes in much of the proteome of *E. huxleyi* despite the two to three‐fold reduction in growth rate and large physiological changes (e.g. cellular biomass and C, N and P), due to nutrient limitation. Similarly, relatively small changes in the abundance of proteins involved in many key pathways were observed in our previous study of acclimation of *E. huxleyi* to irradiance (McKew *et al*., 2013a, 2013b) and that of Jones and colleagues (2013) to elevated CO_2_ (1390 p.p.m. versus 390 p.p.m.). In contrast to the stability of most of the proteome to a range of abiotic factors, abiotic limitation or stress can induce large changes in subsets of proteins that are involved in counteracting the limitation or stress. For example, *E. huxleyi*'s response to high irradiance involved increases in proteins involved in photoprotection at the expense of those involved in light harvesting (McKew *et al*., 2013b). In contrast, in the current study, we found that acclimation of *E. huxleyi* to nutrient limitation was most evident in marked increases in the abundance of proteins involved in inorganic nutrient transport and both the scavenging and internal remobilization of organic forms of N and P (e.g. C‐N hydrolases, urea and amino acid/polyamine transporters and AP). Nonetheless, these proteins that were upregulated under N or P limitation accounted for only 1.7% and 5.7% of the total spectral counts respectively. This highly targeted reorganization of the proteome towards scavenging of DON and DOP gives unique insight into how *E. huxleyi* has found its niche to bloom in highly oligotrophic surface waters.

## Experimental procedures

### Culture conditions

Cultures of the calcifying strain *E. huxleyi* 1516 were grown in filter sterilized (0.2 μm) artificial seawater enriched seawater, artificial water (ESAW) (Berges *et al*., 2001) with bicarbonate amended to 3 mM and enriched with f/8 vitamins and trace metals (Guillard and Ryther, 1962) and the addition of 1 nM selenium. Inorganic N and P additions were altered in the different treatments. Nutrient‐replete media contained 150 μM NH_4_NO_3_ and 20 μM PO_4_^3−^ (N : P = 15 : 1), N‐limited media contained 25 μM NH_4_NO_3_ and 20 μM PO_4_^3−^ (N : P = 2.5 : 1), whereas the P‐limited media contained 150 μM NH_4_NO_3_ and 7 μM PO_4_^3−^ (N : P = 43 : 1). Stock cultures used as inocula were pretreated with antibiotics to remove any contaminating bacteria. The cultures were grown in 3 l cylindrical borosilicate glass vessels and maintained at a constant temperature of 18°C. The cultures were gently and continuously stirred with a magnetic stirrer bar and aerated (100 cm^3^ min^–1^) via a borosilicate glass gas distribution tube (8 mm diameter, porosity 1; Fisher Scientific, Loughborough, UK). The cultures were illuminated with cool‐white fluorescent tubes (Lumilux CoolWhite, OSRAM, Munich, Germany) on a light : dark cycle of 16 : 8 h at a PFD of 300 μmol photons m^–2^ s^–1^ (measured in the centre of the culture vessels while containing distilled water with a 4π Biospherical Instruments QSL100 light sensor, San Diego, CA, USA).

Nutrient‐replete cultures were maintained in exponential steady‐state growth phase at cell concentrations of approximately 6 × 10^5^ ml^–1^ by continuous dilution. In the nutrient‐limited cultures, the dilution rate was reduced to achieve specific growth rates that were two to three times lower than under nutrient‐replete conditions. The cells were grown into steady‐state nutrient limitation (confirmed by reduced growth rates and no measurable inorganic N or P in respective vessels) and maintained through 10 generations under these conditions before sampling. Four replicate cultures were grown for each treatment, and all were sampled 4 h into the light cycle. Cultures were monitored daily for cell abundance and to confirm the absence of bacterial contaminants by direct counts with an improved Neubauer hemocytometer (Fisher Scientific) and cell size with a Coulter Counter Z2 particle counter (Beckman Coulter, High Wycombe, UK). Growth rates were calculated from the cell counts and dilution rates as described previously (Brading *et al*., 2011). Residual dissolved NO_3_^−^ and PO_4_^3−^ in the culture medium were measured daily after filtering (0.2 μm), using the methods of Collos and colleagues (1999) and Solorzano and Sharp (1980) respectively.

### Particulate composition

Samples for determination of POC, PIC, and particulate nitrogen, particulate phosphorus, chlorophyll a, DNA, RNA and protein quantification were collected and processed as described in McKew and colleagues (2013a).

#### Lipid analysis

Cells were filtered onto MF300 glass microfibre filters (Fisher Scientific) and flash‐frozen in liquid N_2_ until extraction. Total lipids were extracted from cells by a modified Bligh and Dyer (1959) method in 9.5 ml of high‐performance liquid chromatography (HPLC) grade methanol : chloroform : H_2_O (10 : 5 : 4 v\v\v) and sonicated for 30 min. A further 2.5 ml of chloroform and 2.5 ml of H_2_O was added (final ratio 10 : 10 : 9) and the extract centrifuged (700 *g*). The lower organic layer was to be separated and dried under N_2_ at 37°C. Lipid fractions were separated using 3 ml Supelco Discovery DSC‐Si Solid Phase Extraction cartridges (Sigma‐Aldrich, St Louis, MO, USA) with 0.5 g sodium sulfate added to the top of the cartridges as a drying agent. Neutral lipids were eluted with 10 ml chloroform, glycol lipids with 10 ml of acetone and phospholipids with 10 ml of methanol. All lipid fractions were evaporated to dryness under N_2_ at 37°C. Derivatization was performed by mild alkaline methanolysis (Dowling *et al*., 1986) by reconstituting dried lipids in 1 ml HPLC grade toluene : methanol (1 : 1 v/v dried on sodium sulfate), adding 1 ml of 0.2 M methanolic potassium hydroxide, gently mixing and incubated at 37°C for 30 min. The reaction was stopped with 0.25 ml of 1 M acetic acid. Of HPLC grade hexane : chloroform (4 : 1 v/v), 5 ml was added, sonicated for 30 min, centrifuged to separate the two phases, and the aqueous lower layer was removed and discarded. Of 0.3 M sodium hydroxide, 3 ml was added and the extract filtered via sodium sulfate‐packed glass syringes, and then evaporated to dryness under N_2_ at 25°C. The dried fatty acid methyl esters (FAMEs) were reconstituted with 200 μl of hexane (HPLC grade) containing an alpha‐cholestane injection standard. Fatty acid methyl esters were separated and analysed by a splitless injection of 1 μl onto a Thermo Trace gas chromatograph (injector temp 270°C) coupled to a Thermo DSQ mass spectrometer (transfer line 270°C, ion source 225°C) using a Supelco SP‐2380 fused silica capillary column (30 m length, 0.25 mm ID, 0.2 μm film; Sigma‐Aldrich). Helium was used as the carrier gas (1 ml min^–1^), and the FAMEs were separated by using a temperature programme, starting at 60°C for 1 min increasing at 5°C min^–1^ to 160°C, followed by 10°C min^–1^ to 240°C and 25°C min^–1^ until reaching 270°C. All peak areas were normalized to the injection standard, and the FAMEs were identified and quantified against Supelco FAME analytical standards (Sigma‐Aldrich) and by comparison of ion spectra against the NIST Atomic Spectra Database.

#### Proteomic analysis

Cells were collected from 300 ml of culture by vacuum filtration onto Whatman 3 μm pore polycarbonate filters (GE Healthcare, Little Chalfont, UK), which were flash‐frozen and stored in liquid N_2_ until proteomics analysis. Proteins were extracted, digested with trypsin, the constituent tryptic peptides concentrated and analysed by electrospray‐ionization tandem mass spectrometry on a hybrid high‐resolution LTQ/Orbitrap Velos instrument (Thermo Scientific, Hemel Hempstead, UK) using 2 μg peptides per injection onto 15 cm long pulled‐tip nanocolumn, as detailed in McKew and colleagues (2013b). The resulting MS/MS raw data files were converted to mzXML format using the ReAdW programme and uploaded onto the LabKey server. The open‐source search engine x! tandem was used for protein identification and acquisition of spectral count data. The primary statistical evaluation and filtering of the protein and spectral count data was performed using the PeptideProphet and ProteinProphet programmes (Nesvizhskii *et al*., 2003) integrated into the LabKey CPAS (Computational Proteomics Analysis System version 2.2). Peptides and proteins were filtered at 0.3% false discovery rate to obtain the final dataset. Proteins were quantified by counting the number of MS/MS spectra matched to corresponding proteins, and all counts were normalized to the total number of spectral counts. Each of the four biological replicates for each treatment was re‐injected and analysed three times (technical replicates) for higher confidence of the spectral counts for each biological replicate (i.e. the spectral counts assigned to each biological replicate for any individual protein is the mean of three technical replicates). Uniprot and the *E. huxleyi* 1516 genome (Read *et al*., 2013) databases were used for the identification of proteins.

## Supporting information


**Table S1.** Normalized spectral counts for all detected proteins (each biological replicate presented is the mean of three technical replicates).
